# Pre-Courtship Behavior of *Proholopterus chilensis* (Coleoptera: Cerambycidae) in a *Nothofagus obliqua* (Nothofagaceae) Forest

**DOI:** 10.3390/insects16080847

**Published:** 2025-08-15

**Authors:** Diego Arraztio, Amanda Huerta, Ramón Rebolledo, Americo Contreras, Tomislav Curkovic

**Affiliations:** 1 Programa de Doctorado en Cs. Silvo-Agropecuarias y Veterinarias, Universidad de Chile, Santiago 8820808, Chile; dsarrazt@uchile.cl; 2 Facultad de Ciencias Forestales y de la Conservación de la Naturaleza, Universidad de Chile, Santiago 8820808, Chile; ahuerta@uchile.cl; 3 Facultad de Ciencias Agropecuarias y Medio Ambiente, Universidad de La Frontera, Temuco 4780000, Chile; ramon.rebolledo@ufrontera.cl; 4 Facultad de Ciencias Agronómicas, Universidad de Chile, Santiago 8820808, Chile; a.contreras@uchile.cl

**Keywords:** female calling, male-oriented flight, hasty walking behavior

## Abstract

The pre-courtship behavior of *Proholopterus chilensis* on *Nothofagus obliqua* trees was recorded for the first time, documenting a putative female “calling” behavior and the consequent male “oriented search,” enabling the description of behavioral units, sequences, frequencies, and degrees of stereotypy. Post-resting activity in both sexes began with walking and grooming during the first hour of the scotophase, following a period of daytime inactivity. Subsequently, females extended their ovipositor both horizontally and vertically, alternating between contact with the tree substrate and, simultaneously, walking the surface (=putative calling behavior), while males became active, extending their antennae perpendicularly to the longitudinal body axis. In response to the putative call, males exhibited oriented flight and hasty walking that followed the trajectory and direction previously taken by females on the substrate, ultimately leading to their location. Statistical analysis of behavioral sequences and the stereotypy index indicated that both behaviors were non-random and partially stereotyped. These findings are consistent with previous chemical analyses of female aerations and terminalia performed by our research group, which identified semiochemicals likely functioning as long-range sex pheromones guiding males to the vicinity of the female’s tree, as well as potential trail pheromones facilitating close-range localization. This dual signaling system seems necessary to the high mobility displayed by females during calling behavior, characterized by frequent and extended bidirectional vertical walks along the trunk, which may enhance signal dispersal and/or reduce predation risk. If confirmed, this would represent a novel sexual encounter mechanism within Cerambycidae.

## Simple Summary

Sexual behaviors in insects differ between sexes: while one sex typically selects the mating partner, the other competes with conspecifics for access to copulation. The characterization of pre-courtship behaviors is of interest both from a basic biological perspective to understand reproductive strategies and, from an applied view, for their potential applications. Identifying postures, behavioral sequences, and interactions between sexes associated with the emission and detection of semiochemicals can aid in developing specific, effective, and environmentally safe management techniques. In this study, we describe and quantitatively analyze the calling behavior of females, the consequent oriented search response of males, and the interaction between sexes in *Proholopterus chilensis*, a native longhorn beetle of Southern Chile that has become an important pest of *Nothofagus obliqua*. Our findings indicate that both behaviors are not random, providing insight into the unique mechanism of sexual encounter in this species, and contributing to the definition of conditions to capture the semiochemicals involved, which, eventually, could serve for the development of pheromone-based monitoring and control tools.

## 1. Introduction

Pre-courtship behaviors in animals typically follow non-random and stereotyped patterns in each sex, and their interaction eventually leads to copulation. These behaviors are mediated by specific visual, acoustic, or chemical cues produced by one sex (usually the female) and detected and followed by the other (usually the male) [1]. This sex-specific investment in reproductive resources allows females to reduce associated risks and focus their efforts on egg production, signaling, and mate selection. In contrast, males must actively search for females, exposing themselves to various dangers (e.g., predation) and competing with conspecific males to ensure reproductive success [2,3]. These behaviors are referred to, respectively, as “calling” (performed by the signaling sex) and “oriented searching” toward the emitting source by the receiving sex [4]. Their characterization is crucial for understanding reproductive strategies [5] and for determining the optimal conditions for capturing semiochemicals (e.g., pheromones) involved in these interactions [6], which, once identified, might be used to develop specific, safe, and effective management strategies for pest Cerambycidae species [7].

Evidence within this family reveals two mutually exclusive calling strategies: (i) the male performs behavioral steps that facilitate the production and effective release of an aggregation sex pheromone attracting both sexes [8,9], which is the most commonly reported mechanism in this taxon [10,11]; or (ii) the female adopts specific postures and maneuvers to emit a sex pheromone that attracts only males [12,13]. The latter is rare and has only been documented in a few species within the subfamilies Prioninae and Lepturinae and even fewer cases in Cerambycinae [14]. Calling behavior in cerambycids occurs under specific exogenous (e.g., temperature) and endogenous (e.g., age) conditions, typically following a circadian rhythm. These parameters must be clearly defined for behavioral studies [6,15]. The oriented “searching” behavior of males in response to female-emitted pheromones has also been infrequently reported in Cerambycidae (e.g., [16,17]), largely due to the predominance of aggregation pheromones as the main pre-courtship strategy. In such cases, encounters often occur on inflorescences or fallen logs as individuals move around the site [18,19] or through active searching by attracted individuals [20].

*Proholopterus chilensis* (Blanchard) (Coleoptera: Cerambycidae: Cerambycinae, though its taxonomic status at the subfamily level remains debated [21]) is a wood-boring species endemic to Chile and Argentina, which causes severe damage in forests and plantations of *Nothofagus obliqua* (Mirb.) Oerst. (Nothofagaceae) [22]. The larval stage develops exclusively in live trees of *N. obliqua*, *Nothofagus alpina* Poepp. et Endl., and *Nothofagus dombeyi* (Mirb.) Oerst. (Nothofagaceae). The female oviposits on the bark, eggs hatch in about a month, and their larvae develop in two years, boring extensive galleries in the trunks without killing the tree but severely affecting the quality of the valuable wood in commercial farms. The last instar larva builds a pupal chamber connected to the outside by an orifice plugged with wood chips, which the adult later removes upon emergence. The pupae are found between July and December, and adults emerge between November and February [23], depending on the localities. Our research group has conducted studies aimed at describing the sexual behavior and identifying the semiochemicals that mediate male–female interactions in *P. chilensis* [15,24] to develop rational management strategies. The present study contributes to this effort by describing and analyzing, for the first time, the pre-courtship behaviors of *P. chilensis*, including female calling, the corresponding male-oriented search toward the signal source, and male–female interactions under both indoor and field conditions.

## 2. Materials and Methods

### 2.1. Collection, Taxonomic Identification, and Pre-Experimental Handling of P. chilensis

**Figure 1:**
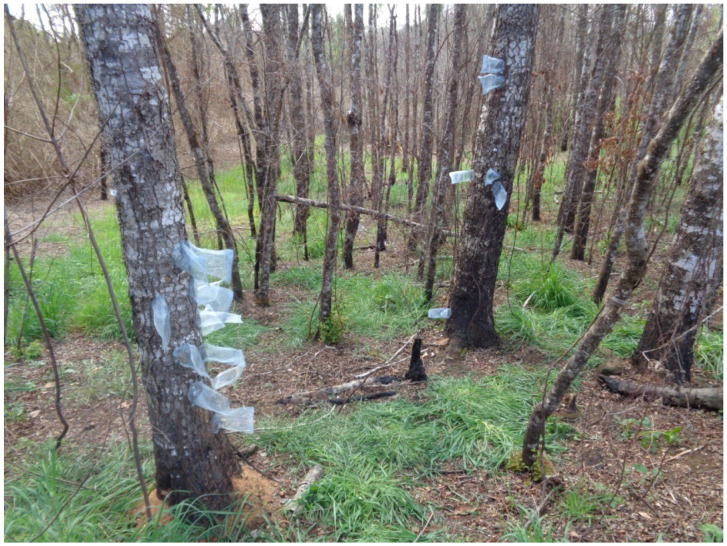
Secondary growth forest of *Nothofagus obliqua* at Maquehue, La Araucanía Region, Chile. Two oaks showing emergence cages and one tree (bottom left corner) with accumulation of sawdust at base of trunk, produced by *P. chilensis* larvae.

Specimens of *Proholopterus chilensis* were collected from secondary growth forests of *Nothofagus obliqua* in Maquehue (−38.8364, −72.6941; La Araucanía Region, Figure 1) and Llifén (−40.1978, −72.2598; Los Ríos Region), Chile, during the spring–summer seasons from 2019 to 2024.

**Figure 2:**
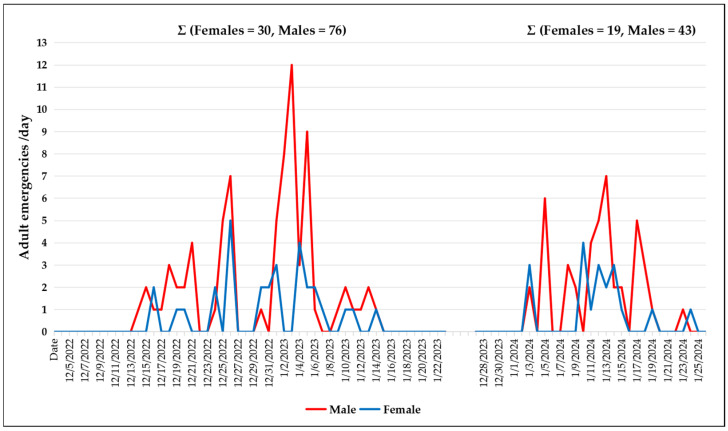
Daily (month/day/year) and seasonal total records (∑) of male and female *P. chilensis* emergences from cages installed on 23 October 2022 (**left**) and on 3 December 2023 (**right**) in Llifén, Los Ríos Region, Chile.

Insects were collected using emergence cages as described previously [15], which allowed for the capture, directly from trunks, of newly emerged, i.e., virgin individuals (“reared”), of known age. In Llifén, daily records of reared adults were kept during the 2022–2023 and 2023–2024 seasons (Figure 2).

Species identification and sexing were performed based on morphological characters described in other works [25,26]. Female genital morphology, referenced in the text, follows the terminology of [27,28]. Following emergence, individuals were kept separately in cages under indoor conditions and fed as described in [15] until their experimental use. Males aged 0–7 days and females aged 0–9 days were used in behavioral studies, corresponding to their active field lifespan [15]. Due to quarantine restrictions in the 2019–2020 season that prevented fieldwork during the COVID-19 pandemic, behavioral observations were conducted indoors in Santiago (−33.4475, −70.6737; Metropolitan Region) using individuals shipped overnight (~8 h transit) by courier from Maquehue. From 2021 to 2024, behavioral observations were conducted indoors and in the field in a stand of *N. obliqua* in Llifén. Individuals used in all observations were collected from either Llifén (20 females, 14 males) or Maquehue (16 females, 10 males). Additionally, during the adult emergence period of *P. chilensis*, emergence cages on trunks were regularly monitored, and naturally occurring wild individuals were observed, both day and night.

### 2.2. Pre-Courtship Behavioral Sequence Experiments in P. chilensis

**Figure 3:**
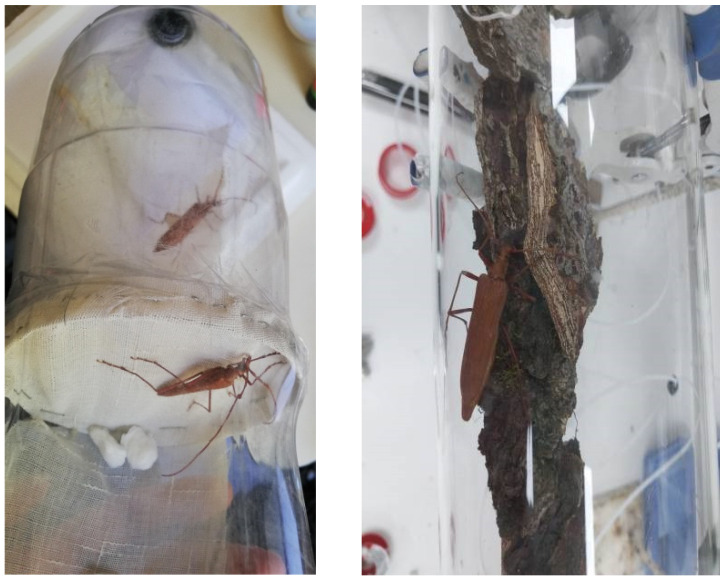
Plastic cylinder arena (**left**) used for *Proholopterus chilensis* behavioral observations indoor showing male (closer) and female (further) separated by piece of cotton mesh; Head Space Dynamic glass chamber with *P. chilensis* female on *N. obliqua* bark (**right**).

Pre-courtship behavioral sequences of *P. chilensis* were recorded by pairing one virgin male and one virgin female at a time. Individuals were used only once, not previously exposed to the opposite sex or manipulated on the day of testing and maintained under proper feeding and environmental conditions [15] to avoid stress. Due to the relative scarcity of individuals, copulation was prevented during observations using physical barriers or by capturing individuals immediately before mounting. This ensured they remained virgin and behaviorally attractive for reuse (e.g., for aereations conducted in parallel assays), though it limited full observation of courtship and copulation behaviors. Three experimental methodologies were used: (I) As described in [15], used in Santiago during the 2019–2020 season (*n* = 6 couples), and in Llifén between 2021 and 2024 (*n* = 18 couples), where pieces of *N. obliqua* trunk were added to the arena floor (Figure 3); (II) During the 2023–2024 season, an open emergence cage (as described in [15]; see Figure 1) containing one virgin female (*n* = 12 replicates) was placed at the base of an *N. obliqua* tree; once the female began moving on the trunk, displaying putative “calling”, a male (*n* = 8 replicates) was released downwind, on a separate tree (2–3 m away) at a height of ca. 1.5 m on the trunk, in a cleared area suitable for flight and field observations; each couple interaction was observed for up to 30 min. If, within that time, the male did not respond with oriented walking or flight, the female was replaced (22% of the individuals tested). If no male response was observed after three different females were tested, the male was replaced (11% of the individuals); a different tree was used for each male or female tested in field trials. The wind speed was recorded using a hot wire anemometer (Extech Instruments, Knoxville, TN, USA). (III) In Llifén (2023–2024), virgin females were individually released at the base of an *N. obliqua* tree; a plastic mesh barrier (“roof”) was placed at ~2 m height around the trunk to prevent upward escape; females ascended the trunk until reaching the barrier, then descended to ~10 cm from the ground, and re-ascended, repeating this cycle several times. Upon reaching the barrier one last time, females made a directional turn or decision (left or right) and remained stationary on the edge of the roof. Then, a male was released in the same manner as the female. Male trajectory and orientation were recorded. The time between female “directional decision” and male release was less than a minute. Behavioral observations using methodologies I and II were conducted between 19:00 and 06:00 over 32 nights. For methodology III, observations were conducted between 21:00 and 06:00 and lasted 27–46 min (from female released to male–female contact). Males did hasty walking (initiated from where females started the putative calling position) until reaching the female in 1–3 min. Recordings were filmed using a video camera (Sony Handycam, HDR-SR10, Japan) with built-in IR. Videos were later analyzed on a laptop (HP Pavilion, 14 screen, Palo Alto, CA, USA) using Adobe Premiere Pro 2020 at 0.8× playback speed to characterize behavioral units and relative frequencies. To avoid disturbing the insects, a red-filtered spotlight (Field Working Lights, HY-9039, 35W Led, Foshan City, China) was used for nocturnal observations. White light was occasionally used for video recording, from which still images were extracted for figures.

### 2.3. Pre-Courtship Behavioral Units and Field Observations of P. chilensis

Behavioral units (steps) comprising post-resting and pre-courtship sequences in males and females were recorded under both indoor and field conditions (methodologies I and II; see Section 2.2). These are listed in Table 1. Only observable (i.e., visible without magnification), discrete (with clearly identifiable start and end points), and homogeneous behavioral units (as defined in [29]) were included. The duration of each step and the time of night when they occurred were extracted from video recordings.

**Figure 4:**
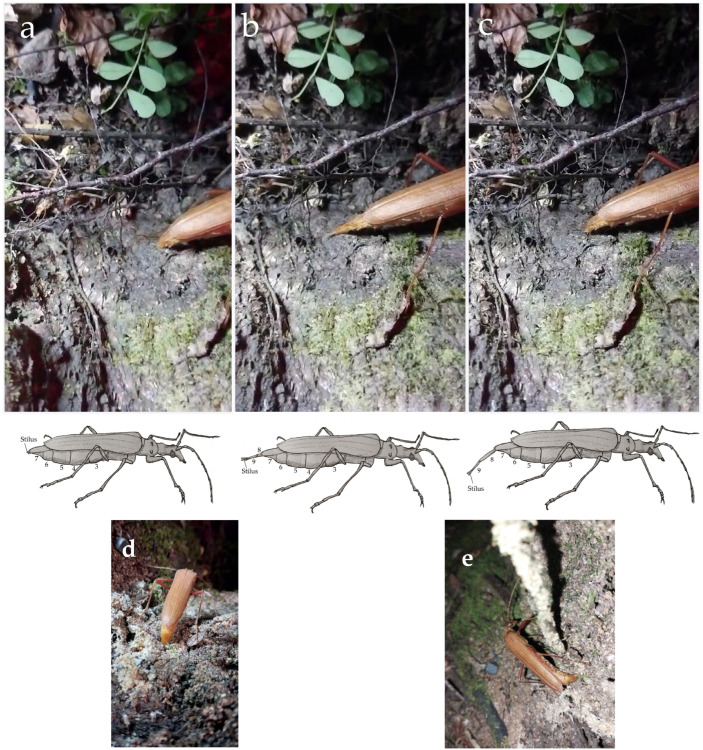
Putative calling sequence in a *P. chilensis* female and possible marking of the path followed on the trunk of an *N. oblique* tree: (**a**) ovipositor not projected; (**b**) projection of telescopic segments and *styli* parallel to the substrate; (**c**) ovipositor bent downward and lightly touching the substrate (“brushstroke”). Below pictures (**a**) through (**c**), there are drawings showing details of the structures projected during every step, including the number of the abdominal sternum. Oviposition pictures (**d**,**e**) presented for comparison with calling.

Figure 4 presents a sequence of photographs captured from video footage, showing the projection of the ovipositor (putative calling phase). An image of oviposition is included in Figure 4 to contrast with calling.

**Figure 5:**
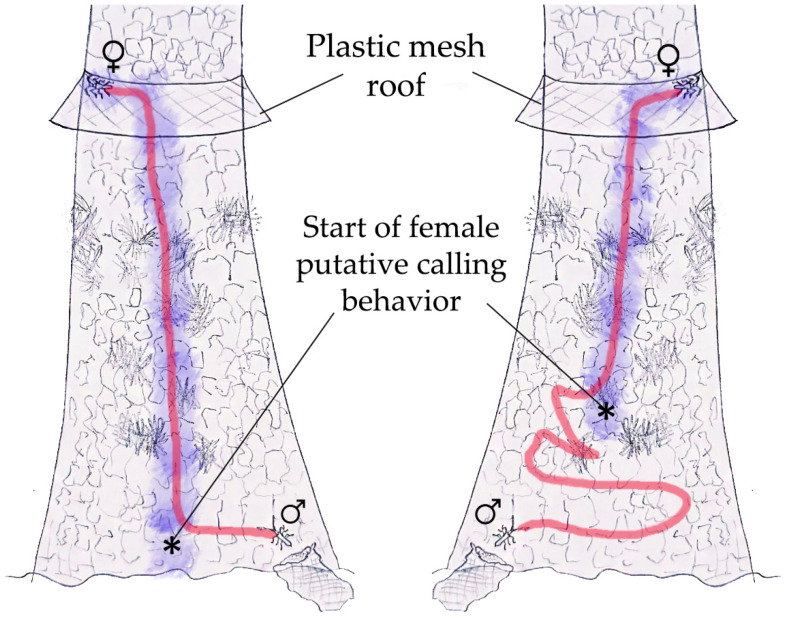
A diagram of the trajectories followed by *P. chilensis* females (purple shadow) and males (red line) on *N. obliqua* trunks before the encounter between sexes at the plastic mesh “roof”. Arrows and asterisks (*) indicate the point where the female, released at the base of the trunk, begins to exhibit calling.

Representative male trajectories recorded under method III are shown in Figure 5. Field observations of cages placed on trunks were conducted every 3–4 h during the day and approximately every hour at night in order to determine the time adult *P. chilensis* were active.

### 2.4. Sequences and Stereotypy of Pre-Courtship Behaviors in P. chilensis

The behavioral data used for analysis come from insects observed for one night only, not reused (to comply with the assumption of independence). Contingency tables of “*i*” (preceding step, in rows) × “*j*” (subsequent step, in columns) cells were built separately for females (Table 2) and males (Table 3).

Each cell indicates the relative frequency of the occurrence of step “*j*” immediately following step “*i*”; in other words, they represent first-order Markov transition frequencies [31]. The chi-square statistic (Σ[(O − E)^2^/E]) of the complete sequence (*χ*^2^_s_) [32] was used to compare the real values (O: observed frequency; E: expected frequency in each cell) with the critical chi-square value (d.f. = [(*i* − 1) × (*j* − 1)]; with 5% significance level) [31]. Self-transitions, i.e., when the preceding step is the same as the following one, were very rare and were not recorded in the contingency tables, following advantages and adjustments suggested by [31,33]. This approach has been used in studies of sexual behavioral sequences in insects [29,34,35], including species of Cerambycidae [6,36]. When the null hypothesis (H_0_: behavioral steps occur independently) was rejected, the same statistic was applied at the cell level (*χ*^2^_c_). When the absolute value of *χ*^2^_c_ > ([*χ*^2^ d.f. = 1; 5% significance level])/R^2^, where R is the repertoire of behavioral steps, exceeded the critical value, the transition from “*i*” to “*j*” significantly differed from random, thus indicating dependence between steps. When *χ*^2^_c_ > 0, step “*i*” may induce step “*j*”, whereas if *χ*^2^_c_ < 0, “*i*” may inhibit “*j*”. The chi-square test was also used to test for independency among categorical variables arranged in 2 × 2 contingency tables (Table 4) to evaluate the relationship between male searching to females projecting their ovipositor (putative calling) and the direction chosen by free-moving females on tree trunks and the direction subsequently followed by released males (methodology III; see Figure 4).

The contingency coefficient (CC) was calculated as described in [32] as a measurement of the association for cross-classification data. The statistical powers for all tests were also calculated. Statistical analyses were performed using Microsoft Office Excel (2021) and R software version 4.4.3 [37]. Additionally, the variability of the behavioral sequences presented in Table 1 and Table 2 was assessed using the stereotypy index (SI), calculated separately for calling (females) and searching (males), according to the following formula:

$$\mathrm{SI}\;=\;\sqrt{\left[\frac{\sum {\left(P_{ij}\right)}^{2}-(\sum P_{ij}{)}^{2}/r_{i}}{\left(1-1/r_{i}\right)}\right]}$$

where 0 ≤ SI ≤ 1. Here, *P_ij_* is the transition probability from behavioral step *i* to each of the subsequent *j*-steps (i.e., the frequency in each cell of row *i* divided by the row total in the contingency table), and *r_i_* is the number of possible transitions from the preceding behavior *i*. When SI = 0, the transitions are highly variable, whereas SI = 1 indicates highly fixed or stereotyped transitions [29]. The SI value obtained for each step was weighted by its relative frequency in the dataset. The sum of these values across all steps in a sequence yielded to the global stereotypy index (GSI), with 0 ≤ GSI ≤ 1, which characterizes the overall stereotypy of the complete behavioral sequence [29], allowing for comparisons with similar studies.

### 2.5. Ethogram of Pre-Courtship Behavior of P. chilensis

**Figure 6:**
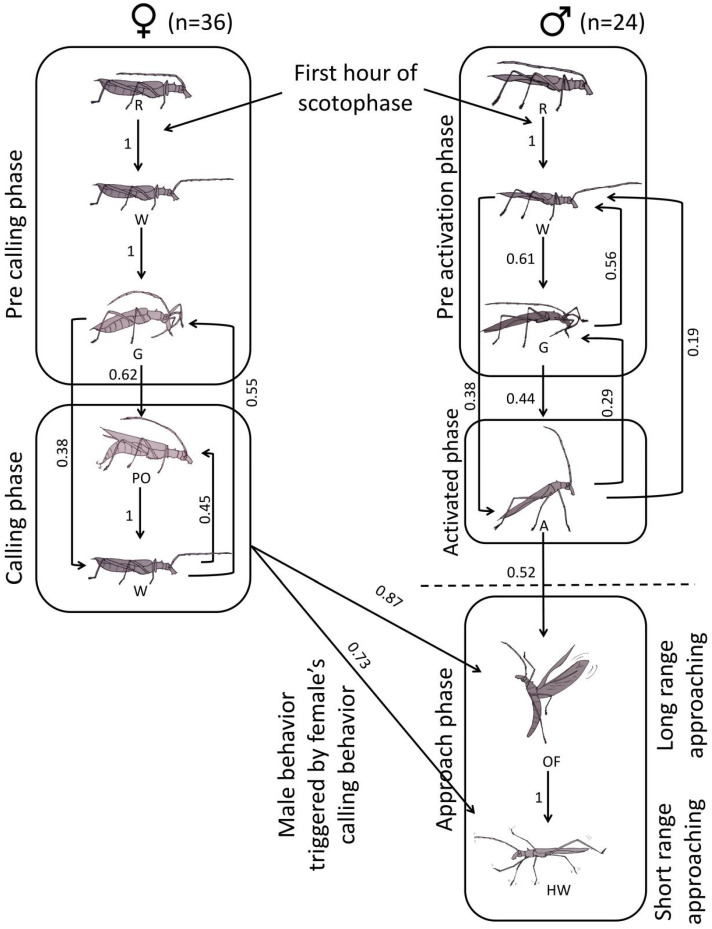
Ethogram of pre-courtship behavioral units in *P. chilensis* males and females. R = rest; W = walk; G = grooming; PO = projection of ovipositor; A = activation; OF = oriented flight; HW = hasty walking.

The complete sequences of behavioral units that constitute the female calling, the male-oriented searching behaviors, and the interactions between sexes are presented in Figure 6. The steps in the sequences are arranged according to their temporal order of occurrence and illustrated with hand-drawn sketches based on the recorded observations. The figures indicate the transition frequencies between pairs of observed steps that fully or partially met the assumptions and constraints of the following analysis [31]. The ethogram displays, using arrows, the possible routes within each sequence and their directionality, as induced by the preceding step. In the final frames of Figure 6, the interactions between the sequences exhibited by both sexes are linked with arrows indicating their frequency and direction, based on observations of male hasty walking toward females (methodology I and III) and male oriented flight behavior (methodology II).

## 3. Results

### 3.1. Field Emergence of P. chilensis

During the various study seasons (2019–2024), 79 females and 183 males were collected using emergence cages; these specimens (reared) were subsequently used in the experimental methodologies described in Section 2.2. In Llifén, individuals emerged over approximately 3–4 weeks during the 2022–2023 and 2023–2024 seasons, from mid-December to mid-January (see Figure 2). This emergence period was significantly shorter than previously reported by [23], although the cited reference likely refers to populations across the entire distribution range of the species in Chile. Emergences in cages showed a degree of protandry and, in both sexes, ceased in a relatively synchronous manner, a pattern previously reported in other species of this family [38]. During field observations of cages placed on trunks, male and female emergences of *P. chilensis* were always observed during the scotophase (*n* = 100 cases) and never during the photophase. In 18 of these newly emerged females (reared), wild males were observed approaching and landing at some distance from them on the same trunk, suggesting that female calling begins on the first day of adult life and orients the males (*n* = 19) to land nearby. These wild males then rapidly walked along the trunk until they reached the cage. A similar behavior was observed when reared females were mounted on interception traps [15]: wild males (*n* = 70) approaching these traps first landed on adjacent trees or nearby shrubs (up to 3 m away), then flew toward the trap, and finally hurried along its surface toward the female. During nighttime observations, seven wild females were found on *N. obliqua* trunks at heights ranging from approximately 0.1 to 2 m. These females performed continuous bidirectional walking (upward or downward) while simultaneously contacting the trunk with the tip of their ovipositor approximately every 5 to 15 cm. Some descended to about 10 cm above the ground and then ascended again, generally following the same route used for the descent. This sequence was observed 2–4 times per female. When females reached heights of approximately 2 m, most were captured to prevent escape; however, a few individuals were observed climbing over 8 m high and subsequently descending roughly along the same “path” used for ascent, covering distances up to ~80 linear m during a single night (between ~30 and ~50 m under methodology I). In response to these wild females, three wild males were observed flying toward the trunk, although they never landed directly on or immediately adjacent to the females. Afterwards, they walked rapidly along the trunk for distances ranging from 1.5 to 8 m, following the route previously taken by the female until reaching her. Females were never observed flying in the field.

### 3.2. Behavioral Units of Proholopterus chilensis in Observation Arenas and in the Field

Males and females of *P. chilensis* reared and observed using methodologies I and II remained at rest (see Table 1) for most of the day, a phase that appears to be regulated by circadian rhythms [39], with no perceptible R, which has been defined as a “sleep” stage in Cerambycidae [40]. The behavioral units developed by *P. chilensis* (using methodologies I, II, and III) are described in Table 1. Using methodology I, it was observed that during the last hour of the scotophase, males and females fed and then walked around inside the arena and groomed themselves without interacting before returning to a resting state at the end of the scotophase. In the field, during the day, individuals were observed fully or partially sheltering beneath the layer of epiphytes covering the trunk. Figure 4 presents pictures and drawings of the putative calling behavior of a *P. chilensis* female on a *Nothofagus obliqua* trunk.

### 3.3. Pre-Courtship Behavioral Sequences and Stereotypy in P. chilensis

The analysis of behavioral sequences (Table 2 and Table 3) allowed us to reject the null hypothesis (H_0_), indicating that there is a dependency between the steps in both females (*χ*^2^ = 493.9; d.f. = 4; *p*-value < 0.00001; CC = 0.78; Cohen index = 0.92; statistical power = 1) and males (*χ*^2^ = 229.1; d.f. = 12; *p*-value < 0.00001; CC = 0.83; Cohen index = 1.40; statistical power = 1) although in the latter case, the assumption of calculated frequencies <5 in no more than 20% of cells [31] was not met for males.

The statistical analysis of categorical variables related to behaviors observed in male–female pairs of *P. chilensis*, considering a critical chi-square value of *χ*^2^ table = 3.841 (*p* = 0.05, d.f. = 1), yielded a *p*-value < 0.00001 in both comparisons. Therefore, the null hypothesis (H_0_) was rejected, indicating dependence between the row and column categories presented in Table 4.

Regarding stereotypy, we obtained GSI = 0.41 in males and GSI = 0.48 in females, indicating an intermediate degree of variability (or fixity) in the behavioral paths followed by the insects in the sequences informed in Table 2 and Table 3. In females, this is due to the relatively even distribution of transition probabilities (P*_ij_* > 0.24) across five effective transitions: the projection of the ovipositor transition (as the preceding step) was followed exclusively by the walk behavior (with grooming never occurring after the projection of the ovipositor). In males, the relatively greater variability in the sequence is attributed to a higher number of effective transitions (*n* = 8), with P*_ij_* > 0.09; the only possible transition after oriented flight is hasty walking, making it completely stereotyped, though it contributed little to the overall GSI. There is no consensus on the GSI values that define the degree of stereotypy in a behavioral sequence. Some authors consider behaviors to be highly stereotyped when GSI ≥ 0.5 for predation behaviors in Araneae [41]. In the case of insect sexual behavior, a sequence has been considered highly stereotyped when GSI > 0.87 [42] or GSI > 0.92 [43] and moderately stereotyped when GSI > 0.76. According to these criteria, the calling behavior in females and the searching behavior in males of *P. chilensis* show relatively medium values of stereotypy compared to that reported in another cerambycid species [6].

Figure 5 describes two situations observed during the trajectory and directional test of the males relative to the route previously followed by the females. In one case, the female began to exhibit the projection of the ovipositor nearly from the base of the trunk (left drawing). In the other case (right one), she initiated the projection of the ovipositor further up after a section where the progress was erratic due to apparent stress, evidenced by stridulation. In the first case, the male, also released at the base of the trunk, immediately began hasty walking and followed a linear path upwards, almost totally overlapping the female’s route. In the right drawing, the male in the first section performed walk, wandering until reaching the point where the female had previously started the projection of the ovipositor, and from there, the male switched to hasty walking, following a relatively linear trajectory. Upon reaching the ceiling, half of the females turned right, and the other half turned left. When males reached the ceiling, they always chose the same direction previously taken by the female. This indicates that the final portion of the male’s search is not random but significantly (*χ*^2^ = 6, d.f. = 1, *p*-value = 0.016) influenced by the female’s prior trajectory and direction.

### 3.4. Ethogram

Figure 6, above the segmented line, presents the ethogram of the behaviors displayed by females (left) and males (right) based on the results obtained with methodologies I and II described in Section 2.2 and the respective transition probabilities calculated from Table 2 and Table 3. Transition probabilities for female–male interactions (below the segmented line) were estimated from all the respective observations obtained using the different methodologies (I, II, and III) throughout the study seasons when, at the same time, the female was/was not performing the projection of the ovipositor and males were/were not performing oriented flight and hasty walking.

## 4. Discussion

Pre-courtship sexual behaviors (“calling” and “oriented search”) in Cerambycidae have been rarely reported, partly due to the cryptic and nocturnal habits of some species, whose adult stage is relatively brief [14], as it also occurs in *P. chilensis*. Calling behavior has mostly been described in species from the subfamilies Cerambycinae, Lamiinae, and Spondylidinae, in which the male typically emits an aggregation sex pheromone that attracts both sexes [10,11,44]. However, this behavior is very rare among females in this family. It has been described only in a few species of Prioninae (whose females, like those of *P. chilensis*, are large and limited in flight capacity [45]), Cerambycinae, and Necydalinae. For example, *Prionus californicus* (Prioninae) females lower their heads and raise the posterior end of the abdomen 20–30° from the horizontal axis while extending their ovipositor and remaining stationary for several minutes [46]; *Anoplophora glabripennis* (Lamiinae) females project and curve the terminalia toward the substrate [47]; and, in *Callisphyris apicicornis* (Necydalinae), diagnostic movements of the legs, elytra, membranous wings, and abdomen along with occasional ovipositor projection have been described [6]. During putative calling, some of these postures and maneuvers were also observed in our study of *P. chilensis* females. Females projected telescoping segments of their ovipositor and alternately and repeatedly rubbed in brief pulses (“brushstrokes”) over the tree trunk (or substrate) as they moved bidirectionally over relatively long distances. Only in response to these female behaviors did *P. chilensis* males initiate the search phase at some distance downwind. The nocturnal habit of *P. chilensis* limited detailed observations of the male-oriented flight toward the females. We observed only the final phase of the flight, no more than 1 m away, showing a pendular-zigzagging (“casting,” [48]) pattern during the last 20–50 cm before landing, with a gradual reduction in amplitude and speed. Males kept their bodies vertical relative to the ground, and the antennae slightly projected forward during this phase. This is similar to what has been described for *C. apicicornis* by [6,49], and we assume that this behavior in *P. chilensis* males is mediated by a long-range sex pheromone. Reared males of *P. chilensis* released downwind from calling females performed oriented flight, never landing on or next to the female, but usually at some distance (0.2–2 m) on the same trunk or a nearby plant. In the latter case, they made short flights to reach the target plant, as previously reported in other species [50]. On the tree where the *P. chilensis* female was located, males landed near the zone she had previously traveled while calling. From there, the males walked toward her position, like reports for *Nadezhdiella cantori* [51] and *Anoplophora malasiaca* [52]. However, in *P. chilensis*, this was a rapid (hasty) walk with exaggerated leg movements consistent with male competition for mates. During this phase, males directed their antennae forward, periodically touching the bark surface. Other cerambycid species have also described such behaviors [5,6,16,53] in response to female volatiles, including long-range sex pheromones.

Our results demonstrate that males always followed the same route and direction previously traveled by calling females on the substrate until reaching them. Given the step-by-step dependence on the pre-courtship behaviors of *P. chilensis* and the level of stereotypy, we conclude that this species develops significant, though variable, pathways during this phase. This suggests that, rather than the sequence alone, different components determine the sexual behavior occurrence, as described in other insects, where behavioral sequences are not random [54].

No studies were found evaluating environmental effects on male activation in cerambycids. However, flight behavior preceding activation has been assessed in several Cerambycidae species, though at lower wind speeds (up to ~5 m/s), in both lab [17,55,56] and field conditions [57]. This suggests that although wind limits flight in *P. chilensis*, it still occurs at relatively high speeds (observed up to 12 m/s) and likely affects activation. Regarding female calling, few studies directly assess wind effects. Other work [6] concluded that calling in *C. apicicornis* females was not affected by field wind speeds below 5 m/s. As for rain, it is a known limiting factor for flight (and presumably for activation) in other cerambycids [58]. However, its effects on male activation or female calling have not been specifically addressed.

The sequence of pre-courtship events observed in *P. chilensis* partially matches that of *Anoplophora glabripennis* [5]. In *A. glabripennis*, the female calls by emitting a long-range pheromone to attract the male from a distance [12]. Our previous studies on *P. chilensis* females also suggest this, as we isolated and identified generic volatile compounds with potential signaling functions [15]. The behavioral observations here also support this, showing an oriented response in downwind males once females enter the putative calling phase. This strategy, rare in Cerambycidae, may be explained by the relatively large size of *P. chilensis* females [26], their limited flight ability, and possibly a male-biased sex ratio (2.3 males per female [15]). This behavior would reduce female risks, allowing greater investment in egg and signal production and enabling mate selection, leaving males to compete and face higher risks (e.g., predation during flight), consistent with mating systems in many animals, including insects [2,3]. Another similarity between both species is the possible presence of a short-range pheromone. In *A. glabripennis*, the male produces a contact pheromone that guides the female during the final approach. In *P. chilensis*, this may also occur, but with the female producing the chemical cue (some candidate compounds have already been identified [15]). Our study supports this behavioral strategy, as males significantly follow a route and direction apparently chemically marked by the female on the bark. However, this represents an atypical mating strategy, as it lacks the usual reversal in communication roles, in which one sex (typically the female) emits a long-range pheromone, and the other (typically the male) follows with a short-range signal [2,12]. To our knowledge, this has not been reported in Cerambycidae. We propose that this highly unusual strategy in *P. chilensis* is driven by the high mobility of females during calling, which may serve to reduce predation risk (e.g., through predator avoidance [59]) and/or create multiple scent plumes to enhance pheromone dispersal and detection (e.g., depending on canopy height [60,61]), thereby improving the chances of males locating the signal source. These hypotheses require further testing in future studies.

## 5. Conclusions

Males and females of *Proholopterus chilensis* become active and engage in pre-courtship behaviors only during the scotophase.

The pre-courtship sequences of both sexes show significant dependence between their constituent steps but exhibit intermediate stereotypy.

Oriented flight and search behavior by the male occurs after the female displays her ovipositor and touches the substrate with it (putative calling).

The final approach occurs when the male walks rapidly over the tree trunk, apparently following a trail previously marked by the female with the distal end of her ovipositor.

If confirmed, this strategy using a female-produced dual pheromone signal would represent an unusual one among insects and, to our knowledge, not previously described in Cerambycidae.

## Figures and Tables

**Table 1:** Pre-courtship behavioral units performed by females and males of *P. chilensis*.

Behavioral Units	Definitions
Rest	Individuals remain immobile and in the same location, with the antennae directed backward and elytra resting over the dorsum of the body from the last hour of the previous scotophase until the second hour of the following one.
Grooming	Individuals bend their antennae with the aid of forelegs, directing them toward the mouthparts passing the medial and apical antennal segments between the mandibles. Both sexes performed this behavior for 3–10 min; females repeated it up to 40 times, while males did so 1 to 6 times, per night.
Walk	Individuals walk slowly, at a constant pace, in a marching-like fashion, with the antennae directed forward or sideways and the legs barely surpassing the height of the elytra, which remains flat over the dorsum of the abdomen. Each walk lasts 1–10 min in males and 5–10 min in females. Females tend to walk continuously, stopping only to groom.
Projection of the ovipositor	Females project segments VIII and IX posteriorly. These telescoping segments form the final part of the ovipositor and remain invaginated at rest. The y-shaped *styli* is located at the distal end of it. During this phase, the female walks slowly and alternately moves the tip of the ovipositor in a particular sequence: first backward (parallel to the substrate), then upward, and finally downward, while briefly dragging the *styli* over the substrate, resembling a “brushstroke.” Each “brushstroke” lasted an average of 7 s and was repeated for 50 to 70 min each night in the absence of males. This female movement and putative marking behavior ceased due to unfavorable environmental conditions (wind, rain), the circadian cycle, or an encounter with a male. During this behavior, there is no ovipositor penetration into the trunk or the epiphyte layer covering the bark surface, nor any change in ovipositor width. After the brushstroke, the ovipositor is retracted back into the abdomen, and the sequence is repeated multiple times throughout the night. During the projection of the ovipositor, when females were on trunks, they placed themselves vertically, with their antennae directed perpendicularly or forward relative to the substrate and the elytra slightly raised. This behavior is assumed to represent the putative calling in *P. chilensis*.
Activation	The male, while stationary, raises their antennae perpendicularly to the longitudinal axis of the body and maintains the position. The male also extends their forelegs forward, lifting the anterior part of the body at a 45° angle relative to the horizontal. Sometimes, the orientation shifts, but the male remains in the same location. This posture can be held for 10–40 min. This behavior was not observed in females.
Oriented flight	The male opens its elytra, unfolds the membranous wings, and takes off; during flight, the body maintains a semi-vertical position relative to the substrate, with the antennae slightly projected forward. The flight is relatively linear when the male is released 2–3 m from the female but becomes pendular-zigzagging (casting) near (final 20–40 cm) the tree where she is located before landing. This behavior was not observed in females.
Hasty walking	The male lands on the plant where the calling female is located and projects its antennae forward, touching the substrate with their tips while walking rapidly, lifting its legs exaggeratedly above the dorsal level of the elytra. Males follow the route previously traveled by the female. This behavior was not observed in females.
Oviposition	Stationary females project the terminal ovipositor segments posteriorly for several minutes and insert them into crevices in the wood or within the epiphyte layer covering the tree trunk. During this phase, the flow of eggs (or ovules) significantly increases the ovipositor width.

For morphological features, see references [27,30].

**Table 2:** Relative frequencies between behavioral steps (previous in rows, next in columns) comprising pre-courtship sequences exhibited by *P. chilensis* females (*n* = 36).

Next step (→) Previous Step (↓)	Grooming	Walk	Projection of the Ovipositor	∑ *→
Grooming	**	35	57	92
Walk	71	**	59	130
Projection of the ovipositor	0	59	**	59
∑↓	71	94	116	∑total: 281

Down arrows refer to “previous step”; straight arrows refer to “next step”; *: sums (rows, columns, total); **: auto-transitions not included.

**Table 3:** Relative frequencies between behavioral steps (previous in rows, next in columns) comprising pre-courtship sequences exhibited by *P. chilensis* males (*n* = 24).

Next Step (→) Previous Step (↓)	Grooming	Activation	Walk	Oriented Flight	Hasty Walking	∑ *→
Grooming	**	15	19	0	0	34
Activation	6	**	4	11	0	21
Walk	19	12	**	0	0	31
Oriented flight	0	0	0	**	12	12
∑↓	25	27	23	11	12	∑total: 98

Down arrows refer to “previous step”; straight arrows refer to “next step”; * Sums (rows, columns, total); **: auto-transitions not included.

**Table 4:** Chi-square statistics for male behaviors of *P. chilensis* and their dependence on occurrence (or not) of projection of ovipositor in females.

Categories in Rows (Behaviors)	Categories in Columns (Behaviors)	Couples (♀♂) (*n*)	*χ*^2^ *	CC **	CI ***	SP ****
♀ does/does not project the ovipositor	♂ does/does not perform oriented flight	8	28.48	0.79	0.81	0.99
	♂ does/does not perform hasty walking	12	57.735	0.84	0.73	0.99

*: calculated *χ*^2^; **: contingency coefficient; ***: Cohen index; ****: statistical power.

## Data Availability

The raw data supporting the conclusions of this article will be made available by the authors on request.

## References

[1] Matthews R.W., Matthews J.R. (2010). Insect Behavior.

[2] Alexander R.D., Marshall D.C., Cooley J.R., Choe J.C., Crespi B.J. (1997). Evolutionary perspectives in insect mating. The Evolution of Mating Systems in Insects and Arachnids.

[3] Brown W.D., Crespi B.J., Choe J., Choe J.C., Crespi B.J. (1997). Sexual conflict and the evolution of mating systems. The Evolution of Mating Systems in Insects and Arachnids.

[4] Wyatt T.D. (2008). Pheromones and Animal Behavior: Communication by Smell and Taste.

[5] Xu T., Teale S.A. (2021). Chemical ecology of the Asian longhorn beetle, *Anoplophora glabripennis*. J. Chem. Ecol..

[6] Curkovic T., Ferrera C. (2012). Female calling and male flight orientation and searching behaviors in *Callisphyris apicicornis*: Evidence for a female-produced sex attractant pheromone. Cienc. E Investig. Agrar..

[7] Allison J.D., Borden J.H., Seybold S.J. (2004). A review of the chemical ecology of the Cerambycidae (Coleoptera). Chemoecology.

[8] Mitchell R.F., Graham E.E., Wong J.C., Reagel P.F., Striman B.L., Hughes G.P., Hanks L.M. (2011). Fuscumol and fuscumol acetate are general attractants for many species of cerambycid beetles in the subfamily Lamiinae. Entomol. Exp. Appl..

[9] Mitchell R.F., Reagel P.F., Wong J.H.C., Meier L.R., Diaz Silva W., Mongold-Diers J., Millar J.G., Hanks L.M. (2015). Cerambycid beetle species with similar pheromones are segregated by phenology and minor pheromone components. J. Chem. Ecol..

[10] Lacey E.S., Ray A.M., Hanks L.M. (2007). Calling behavior of the cerambycid beetle *Neoclytus acuminatus acuminatus* (F.). J. Insect Behav..

[11] Lemay M.A., Silk P.J., Sweeney J. (2010). Calling behavior of *Tetropium fuscum* (Coleoptera: Cerambycidae: Spondylidinae). Can. Entomol..

[12] Wickham J.D., Xu Z., Teale S.A. (2012). Evidence for a female-produced, long-range pheromone of *Anoplophora glabripennis* (Coleoptera: Cerambycidae). Insect Sci..

[13] Hanks L.M., Millar J.G. (2016). Sex and Aggregation-Sex Pheromones of Cerambycid Beetles: Basic Science and Practical Applications. J. Chem. Ecol..

[14] Cervantes D.E., Hanks L., Lacey E.S., Barbour J.D. (2006). First documentation of a volatile sex pheromone in a longhorned beetle (Coleoptera: Cerambyicidae) of the primitive subfamily Prioninae. Ann. Entomol. Soc. Am..

[15] Arraztio D., Huerta A., Quiroz A., Aniñir W., Rebolledo R., Curkovic T. (2024). Factors to Male-Female Sex Approaches and the Identification of Volatiles and Compounds from the Terminalia of *Proholopterus chilensis* (Blanchard) (Coleoptera: Cerambycidae) Females in *Nothofagus obliqua* (Mirb.) Oerst. (Nothofagaceae) Forests in Chile. Insects.

[16] Kobayashi H., Yamane A., Iwata R. (2003). Mating behavior of the pine sawyer, *Monochamus saltuarius* (Coleoptera: Cerambycidae). Appl. Entomol. Zool..

[17] Keena M.A. (2018). Factors that influence flight propensity in *Anoplophora glabripennis* (Coleoptera: Cerambycidae). Environ. Entomol..

[18] Iwabuchi K. (1982). Mating behavior of *Xylotrechus pyrrhoderus* Bates (Coleoptera: Cerambycidae) I. Behavioral sequences and existence of the male sex pheromone. Appl. Entomol. Zool..

[19] Ray A.M., Ginzel M.D., Hanks L.M. (2009). Male *Megacyllene robiniae* (Coleoptera: Cerambycidae) Use Multiple Tactics When Aggressively Competing for Mates. Environ. Entomol..

[20] Kiriyama S., Iwata R., Fukaya M., Hoshino Y., Yamanaka Y. (2018). Mating behavior of *Rosalia batesi* (Coleoptera: Cerambycidae) is mediated by male-produced sex pheromones. Insects.

[21] Švácha P., Lawrence J.F., Leschen R.A.B., Beutel R.G. (2014). Volume 3: Morphology and Systematics (Phytophaga). Handbook of Zoology: Arthropoda: Insecta: Coleoptera, Beetles.

[22] Barriga J.E., Curkovic T., Fichet T., Henríquez J., Macaya J. (1993). Nuevos antecedentes de coleópteros xilófagos y plantas hospederas en Chile, con una recopilación de citas previas. Rev. Chil. De Entomol..

[23] Baldini A., Alvarado A. (2008). Manual de Plagas Y Enfermedades Del Bosque Nativo en Chile.

[24] Curkovic T., Arraztio D., Huerta A., Rebolledo R., Cheuquel A., Contreras A., Millar J.G. (2022). Generic pheromones identified from northern hemisphere Cerambycidae (Coleoptera) are attractive to native longhorn beetles from Central-Southern Chile. Insects.

[25] Artigas J. (1994). Holopterus chilensis Blanchard, taladrador del roble. Entomología Económica: Insectos de Interés Agrícola, Forestal, Médico Y Veterinario (Nativos, Introducidos Y Susceptibles de Ser Introducidos).

[26] Barriga A. (2021). Caracterización Morfológica Y Morfométrica de Proholopterus Chilensis (Blanchard in Gay, 1851) (Col.: Cerambycidae) Y Sus Daños Sobre Nothofagus Obliqua (Mirb.) Oerst. en la Región de la Araucanía.

[27] Hutcheson J.A. (1980). *Arhopalus ferus* (Coleoptera: Cerambycidae): Structure and function of the female reproductive system. N. Z. J. Zool..

[28] Monné L., Monne A., Wang Q., Wang Q. (2017). General morphology, classification and biology of Cerambycidae. Cerambycidae of the World: Biology and Pest Management.

[29] Haynes K.F., Birch M.C. (1984). Mate-locating and courtship behaviors of the artichoke plume moth, *Platyptilia carduidactyla* (Lepidoptera: Pterophoridae). Environ. Entomol..

[30] Hubweber L., Schmitt M. (2010). Differences in genitalia structure and function between subfamilies of longhorn beetles (Coleoptera: Cerambycidae). Genetica.

[31] Fagen R.M., Young D.Y., Colgan P.W. (1978). Temporal patterns of behaviors: Durations, intervals, latencies, and sequences. Quantitative Ethology.

[32] Ott R.L. (1993). An Introduction to Statistical Methods and Data Analysis.

[33] Colgan P.W., Smith J.T., Colgan P.W. (1978). Multidimensional contingency table analysis. Quantitative Ethology.

[34] Liimatainen J., Hoikkala A. (1998). Interactions of the males and females of three sympatric Drosophila virilis-group species, *D. Montana*, *D. littoralis*, and *D. lummei*, (Diptera: Drosophilidae) in intra- and interspecific courtships in the wild and in the laboratory. J. Insect Behav..

[35] Girling R.D., Cardé R.T. (2006). Analysis of the courtship behavior of the navel orangeworm, *Amyelois transitella* (Walker) (Lepidoptera: Pyralidae), with a commentary on methods for the analysis of sequences of behavioral transitions. J. Insect Behav..

[36] Godínez-Aguilar J.L., Macías-Sámano J.E., Morón-Ríos A. (2009). Notes on biology and sexual behavior of *Tetrasarus plato* Bates (Coleoptera: Cerambycidae), a tropical longhorn beetle in coffee plantations in Chiapas, Mexico. Coleopt. Bull..

[37] R Core Team (2025). R: A Language and Environment for Statistical Computing.

[38] Sarto V., Torras G. (2018). A new alien invasive longhorn beetle, *Xylotrechus chinensis* (Cerambycidae), is infesting mulberries in Catalonia (Spain). Insects.

[39] Royzenblat S., Kulacic J., Friedrich M. (2023). Evidence of ancestral nocturnality, locomotor clock regression, and cave zone-adjusted sleep duration modes in a cave beetle. Subterr. Biol..

[40] Jiang X.L., Ren Z., Hai X.X., Zhang L., Wang Z.G., Lyu F. (2023). Exposure to artificial light at night mediates the locomotion activity and oviposition capacity of *Dastarcus helophoroides* (Fairmaire). Front. Physiol..

[41] Ferreira H., Goncalvez J. (2005). Forrageamento em *Achaearanea cinnabarina* Levi, 1963 (Araneae: Theriididae) e evolucao da caca em aranhas de teia irregular. Biota Neotrop..

[42] Facundo H.T., Linn C.E., Villani M.G., Roelofs W.L. (1999). Emergence, mating, and postmating behaviors of the Oriental beetle (Coleoptera: Scarabaeidae). J. Insect Behav..

[43] Charlton R.E., Cardé R.T. (1990). Behavioral interactions in the courtship of *Lymantria dispar* (Lepidoptera: Lymantriidae). Ann. Entomol. Soc. Am..

[44] Iwabuchi K. (1988). Mating Behavior of *Xylotrechus pyrrhoderus* Bates (Coleoptera: Cerambycidae). VI Mating System. J. Ethol..

[45] Haack R.A., Keena M.A., Eyre D., Wang Q. (2017). Life history and population dynamics of Cerambycidae. Cerambycidae of the World: Biology and Pest Management.

[46] Barbour J.D., Cervantes D.E., Lacey E.S., Hanks L.M. (2006). Calling behavior in the primitive longhorned beetle *Prionus californicus* Mots. J. Insect Behav..

[47] Xu T., Hansen L., Teale S.A. (2019). Female calling behaviour in the Asian longhorned beetle (Coleoptera: Cerambycidae). Can. Entomol..

[48] Cardé R.T. (2021). Navigation along windborne plumes of pheromone and resource-linked odors. Annu. Rev. Entomol..

[49] Curkovic T., Muñoz J. (2011). Characterization of courtship and mating in *Callisphyris apicicornis*: Tool to define the viability to develop management strategies. Agrociencia.

[50] Yasui H., Fujiwara-Tsujii N. (2013). The effects of foods consumed after adult eclosion on the mate-searching behavior and feeding preferences of the white-spotted longicorn beetle *Anoplophora malasiaca* (Coleoptera: Cerambycidae). Appl. Entomol. Zool..

[51] Wang Q., Zeng W., Chen L., Li J., Yin X. (2002). Circadian reproductive rhythms, pair-bonding, and evidence for sex-specific pheromones in *Nadezhdiella cantori* (Coleoptera: Cerambycidae). J. Insect Behav..

[52] Yasui H., Yasuda T., Fukaya M., Akino T., Wakamura S., Hirai Y., Kawasaki K., Ono H., Narahara M., Kousa K. (2007). Host plant chemicals serve intraspecific communication in the white-spotted longicorn beetle, *Anoplophora malasiaca* (Thomson) (Coleoptera: Cerambycidae). Appl. Entomol. Zool..

[53] Sabri M.S., Abdullah F. (2016). Mating behaviour and evidence of a female sex pheromone in *Rytidodera simulans* White (Coleoptera: Cerambycidae). J. Entomol. Res..

[54] Farrell S.L., Andow D.A. (2017). Highly variable male courtship behavioral sequences in a crambid moth. J. Ethol..

[55] Fukaya M., Kiriyama S., Yasui H. (2017). Mate-location flight of the red-necked longicorn beetle, *Aromia bungii* (Coleoptera: Cerambycidae): An invasive pest lethal to Rosaceae trees. Appl. Entomol. Zool..

[56] Jung J.K., Lee C., Jang B., Nam Y. (2025). Effects of Sex, Age, and Body Size on Flight Performance of *Monochamus alternatus* (Coleoptera: Cerambycidae), a Vector of Pine Wood Nematodes, Using Flight Mills. Insects.

[57] Torres-Vila L.M., Mendiola-Diaz F.J., Sánchez-González Á. (2017). Dispersal differences of a pest and a protected C erambyx species (Coleoptera: Cerambycidae) in oak open woodlands: A mark–recapture comparative study. Ecol. Entomol..

[58] Gray B. (1973). Observations on insect flight in a tropical forest plantation: II. Flight activity of *Syllitus* sp. nov.(Col. Cerambycidae). Z. Für Angew. Entomol..

[59] Sheehan T.N., Ulyshen M.D., Horn S., Hoebeke E.R. (2019). Vertical and horizontal distribution of bark and woodboring beetles by feeding guild: Is there an optimal trap location for detection?. J. Pest Sci..

[60] Wermelinger B., Flückiger P.F., Obrist M.K., Duelli P. (2007). Horizontal and vertical distribution of saproxylic beetles (Col., Buprestidae, Cerambycidae, Scolytinae) across sections of forest edges. J. Appl. Entomol..

[61] Miller G.L., Loudon C., Freed S. (2007). Position around a tree: Consequences for pheromone detection. J. Chem. Ecol..

